# Methyl 4-isonicotinamido­benzoate monohydrate

**DOI:** 10.1107/S1600536810024979

**Published:** 2010-07-03

**Authors:** Yang Zhang, Xiao-Li Zhao

**Affiliations:** a Shanghai Key Laboratory of Green Chemistry and Chemical Processes, Department of Chemistry, East China Normal University, 3663 North Zhongshan Road, Shanghai 200062, People’s Republic of China

## Abstract

The title compound, C_14_H_12_N_2_O_3_·H_2_O, synthesized by the reaction of methyl 4-amino­benzoate with isonicotinoyl chloride hydro­chloride, is relatively planar, with the pyridine ring being inclined by 7.46 (7)° to the benzene ring. In the crystal, the methyl 4-isonicotinamido­benzoate mol­ecules are inter­linked by water mol­ecules *via* N—H⋯O, O—H⋯N and O—H⋯O hydrogen bonds, leading to the formation of a double-chain ribbon-like structure.

## Related literature

For the synthesis of methyl 4-amino­benzoate and isonicotinoyl chloride hydro­chloride, see: Margiotta *et al.* (2008 ▶). For the use of such ligands in coordination chemistry, see: Saeed *et al.* (2010 ▶); Kitagawa (2005 ▶). For standard bond distances, see: Allen *et al.* (1987 ▶).
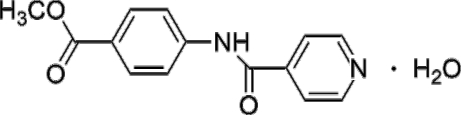


## Experimental

### 

#### Crystal data

C_14_H_12_N_2_O_3_·H_2_O
*M* *_r_* = 274.27
Triclinic, 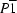

*a* = 6.8836 (3) Å
*b* = 8.8810 (4) Å
*c* = 10.9658 (5) Å
α = 96.062 (1)°
β = 90.896 (1)°
γ = 95.854 (1)°
*V* = 662.91 (5) Å^3^
*Z* = 2
Mo *K*α radiation
μ = 0.10 mm^−1^
*T* = 296 K
0.48 × 0.37 × 0.27 mm

#### Data collection

Bruker SMART 1K CCD area-detector diffractometer
Absorption correction: multi-scan (*SADABS*; Sheldrick, 1996 ▶) *T*_min_ = 0.952, *T*_max_ = 0.973
7689 measured reflections
2318 independent reflections
1968 reflections with *I* > 2σ(*I*)
*R*_int_ = 0.015

#### Refinement

*R*[*F* ^2^ > 2σ(*F* ^2^)] = 0.035
*wR*(*F* ^2^) = 0.102
*S* = 1.06
2318 reflections
181 parameters
3 restraints
H-atom parameters constrained
Δρ_max_ = 0.14 e Å^−3^
Δρ_min_ = −0.16 e Å^−3^

### 

Data collection: *SMART* (Bruker, 2007 ▶); cell refinement: *SAINT* (Bruker, 2007 ▶); data reduction: *SAINT*; program(s) used to solve structure: *SHELXS97* (Sheldrick, 2008 ▶); program(s) used to refine structure: *SHELXL97* (Sheldrick, 2008 ▶); molecular graphics: *SHELXTL* (Sheldrick, 2008 ▶); software used to prepare material for publication: *SHELXTL* and local programs.

## Supplementary Material

Crystal structure: contains datablocks I, global. DOI: 10.1107/S1600536810024979/su2171sup1.cif

Structure factors: contains datablocks I. DOI: 10.1107/S1600536810024979/su2171Isup2.hkl

Additional supplementary materials:  crystallographic information; 3D view; checkCIF report

## Figures and Tables

**Table 1:** Hydrogen-bond geometry (Å, °)

*D*—H⋯*A*	*D*—H	H⋯*A*	*D*⋯*A*	*D*—H⋯*A*
N1—H1*A*⋯O1*W*^i^	0.86	2.10	2.9014 (14)	155
O1*W*—H1*WA*⋯N2^ii^	0.92	1.94	2.8510 (16)	169
O1*W*—H1*WB*⋯O2	0.89	1.96	2.8385 (14)	172

Symmetry codes: (i) 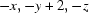; (ii) 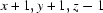.

**Fig. 1.**
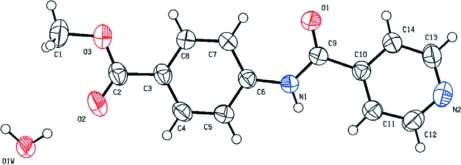
The molecular structure of the title compound, with atom labels and displacement ellipsoids drawn at the 50% probability level.

**Fig. 2.**
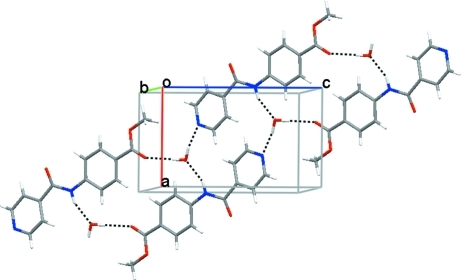
A view of the crystal packing of the title compound, showing one layer of molecules connected by O—H···N, O—H···O and N—H···O hydrogen bonds (dashed lines) [Symmetry codes: A = x+1, y+1, z-1; B = -x, -y+2, -z].

## supplementary crystallographic information

### Comment

The title compound was synthesized as a potential ligand for use in coordination chemistry (Saeed *et al.*, 2010; Kitagawa, 2005). It was synthesized via the reacton of methyl 4-benzoate with isonicotinoyl.HCl and contains both a coordination site [the N atom in the pyridyl ring], and a guest interaction site [the amide group].

The molecular structure of the title compound is illustrated in Fig. 1. The bond distances are normal (Allen *et al.*, 1987), and the molecule is relatively planar with the dihedral angle involving the pyridine and benezene rings being 7.46 (7)°.

In the crystal molecules are connected by hydrogen bonds, with the water molecule H-atoms serving as hydrogen-bond donors and the pyridyl nitrogen and ester oxygen atoms serving as acceptors (Fig. 2 and Table 1). At the same time, the amide nitrogen atom acts as a hydrogen-bond donor and the water oxygen atom as a hydrogen-bond acceptor. In this way a double stranded ribbon-like structure is formed with base vector [11-1].

### Experimental

Methyl 4-aminobenzoate and isonicotinoyl chloride hydrochloride were synthesized using the literature methods (Margiotta *et al.*, 2008). Methy 4-aminobenzoate (3.02 g, 20 mmol), isonicotinoyl chloride hydrochloride (3.56 g, 20 mmol), and K_2_CO_3_ (5.52 g, 49 mmol) were mixed in acetone (100 ml). The mixture was kept at 343 K for 8 h with constant stirring. The white precipitate that formed was filtered off and washed with distilled water and then dried. Colourless block-like crystals, suitable for x-ray analysis, were obtained from a DMF-methanol solution (1:1; v:v) *via* vapour evaporation at room temperature after two weeks.

### Refinement

The water molecule H-atoms were located in a difference Fourier map and were refined with U_iso_(H) = 1.5U_eq_(Ow) and a restrained bond distance of 0.85 (2) Å. The remaining H-atoms were positioned geometrically and refined using a riding model: N-H = 0.86 Å, C—H = 0.93–0.96 Å with *U*_iso_(H) = k × *U*_eq_(N,C), where k = 1.2 for NH, CH and CH_2_ H-atoms, and k = 1.5 for CH_3_ H-atoms.

### Crystal data

**Table:**

C_14_H_12_N_2_O_3_·H_2_O	*Z* = 2
*M**_r_* = 274.27	*F*(000) = 288
Triclinic, *P*1	*D*_x_ = 1.374 Mg m^−^^3^
Hall symbol: -P 1	Mo *K*α radiation, λ = 0.71073 Å
*a* = 6.8836 (3) Å	Cell parameters from 4652 reflections
*b* = 8.8810 (4) Å	θ = 2.3–28.3°
*c* = 10.9658 (5) Å	µ = 0.10 mm^−^^1^
α = 96.062 (1)°	*T* = 296 K
β = 90.896 (1)°	Block, colourless
γ = 95.854 (1)°	0.48 × 0.37 × 0.27 mm
*V* = 662.91 (5) Å^3^	

### Data collection

**Table:**

Bruker SMART 1K CCD area-detector diffractometer	2318 independent reflections
Radiation source: fine-focus sealed tube	1968 reflections with *I* > 2σ(*I*)
graphite	*R*_int_ = 0.015
Detector resolution: 8.192 pixels mm^-1^	θ_max_ = 25.0°, θ_min_ = 1.9°
thin–slice ω scans	*h* = −8→8
Absorption correction: multi-scan (*SADABS*; Sheldrick, 1996)	*k* = −10→10
*T*_min_ = 0.952, *T*_max_ = 0.973	*l* = −13→12
7689 measured reflections	

### Refinement

**Table:**

Refinement on *F*^2^	Primary atom site location: structure-invariant direct methods
Least-squares matrix: full	Secondary atom site location: difference Fourier map
*R*[*F*^2^ > 2σ(*F*^2^)] = 0.035	Hydrogen site location: inferred from neighbouring sites
*wR*(*F*^2^) = 0.102	H-atom parameters constrained
*S* = 1.06	*w* = 1/[σ^2^(*F*_o_^2^) + (0.0526*P*)^2^ + 0.1234*P*] where *P* = (*F*_o_^2^ + 2*F*_c_^2^)/3
2318 reflections	(Δ/σ)_max_ < 0.001
181 parameters	Δρ_max_ = 0.14 e Å^−^^3^
3 restraints	Δρ_min_ = −0.16 e Å^−^^3^

### Special details

**Table:**

Geometry. All esds (except the esd in the dihedral angle between two l.s. planes) are estimated using the full covariance matrix. The cell esds are taken into account individually in the estimation of esds in distances, angles and torsion angles; correlations between esds in cell parameters are only used when they are defined by crystal symmetry. An approximate (isotropic) treatment of cell esds is used for estimating esds involving l.s. planes.
Refinement. Refinement of *F*^2^ against ALL reflections. The weighted *R*-factor wR and goodness of fit *S* are based on *F*^2^, conventional *R*-factors *R* are based on *F*, with *F* set to zero for negative *F*^2^. The threshold expression of *F*^2^ > σ(*F*^2^) is used only for calculating *R*-factors(gt) etc. and is not relevant to the choice of reflections for refinement. *R*-factors based on *F*^2^ are statistically about twice as large as those based on *F*, and *R*- factors based on ALL data will be even larger.

### Fractional atomic coordinates and isotropic or equivalent isotropic displacement parameters (Å^2^)

**Table:**

	*x*	*y*	*z*	*U*_iso_*/*U*_eq_	
N1	0.02099 (15)	0.81758 (12)	0.39503 (10)	0.0431 (3)	
H1A	−0.0956	0.7830	0.3698	0.052*	
N2	−0.34705 (19)	0.52096 (14)	0.70330 (12)	0.0576 (3)	
O3	0.61235 (16)	1.21607 (13)	0.08752 (10)	0.0623 (3)	
O2	0.33143 (18)	1.24867 (15)	−0.00513 (11)	0.0772 (4)	
O1	0.23987 (15)	0.81573 (15)	0.55168 (10)	0.0713 (4)	
O1W	0.30526 (15)	1.33795 (13)	−0.24537 (9)	0.0662 (3)	
H1WA	0.4223	1.3964	−0.2516	0.099*	
H1WB	0.3142	1.3192	−0.1677	0.099*	
C1	0.7154 (3)	1.3157 (2)	0.00895 (17)	0.0722 (5)	
H1B	0.8527	1.3251	0.0290	0.108*	
H1C	0.6685	1.4142	0.0207	0.108*	
H1D	0.6937	1.2740	−0.0751	0.108*	
C2	0.4195 (2)	1.19167 (16)	0.07065 (12)	0.0507 (4)	
C3	0.3244 (2)	1.08944 (15)	0.15573 (11)	0.0440 (3)	
C4	0.1229 (2)	1.05719 (15)	0.14751 (12)	0.0480 (3)	
H4A	0.0521	1.0974	0.0881	0.058*	
C5	0.0271 (2)	0.96663 (15)	0.22619 (12)	0.0462 (3)	
H5A	−0.1079	0.9452	0.2192	0.055*	
C6	0.13107 (19)	0.90658 (14)	0.31652 (11)	0.0397 (3)	
C7	0.3329 (2)	0.93699 (16)	0.32434 (12)	0.0467 (3)	
H7A	0.4041	0.8963	0.3833	0.056*	
C8	0.4280 (2)	1.02781 (16)	0.24441 (13)	0.0479 (3)	
H8A	0.5633	1.0479	0.2501	0.057*	
C9	0.07765 (19)	0.78029 (15)	0.50566 (12)	0.0442 (3)	
C10	−0.07614 (19)	0.69056 (14)	0.57202 (11)	0.0408 (3)	
C11	−0.2737 (2)	0.69375 (16)	0.55336 (12)	0.0483 (3)	
H11A	−0.3201	0.7522	0.4959	0.058*	
C12	−0.4020 (2)	0.60887 (18)	0.62121 (14)	0.0573 (4)	
H12A	−0.5351	0.6136	0.6086	0.069*	
C13	−0.1559 (2)	0.51992 (17)	0.72155 (14)	0.0566 (4)	
H13A	−0.1137	0.4597	0.7790	0.068*	
C14	−0.0171 (2)	0.60287 (16)	0.66023 (13)	0.0515 (4)	
H14A	0.1151	0.6002	0.6778	0.062*	

### Atomic displacement parameters (Å^2^)

**Table:**

	*U*^11^	*U*^22^	*U*^33^	*U*^12^	*U*^13^	*U*^23^
N1	0.0379 (6)	0.0495 (6)	0.0418 (6)	−0.0031 (5)	−0.0043 (4)	0.0129 (5)
N2	0.0608 (8)	0.0573 (7)	0.0527 (7)	−0.0070 (6)	0.0120 (6)	0.0074 (6)
O3	0.0606 (7)	0.0702 (7)	0.0580 (6)	−0.0041 (5)	0.0084 (5)	0.0254 (5)
O2	0.0824 (9)	0.0973 (9)	0.0553 (7)	−0.0071 (7)	−0.0077 (6)	0.0408 (6)
O1	0.0464 (6)	0.1138 (10)	0.0532 (6)	−0.0184 (6)	−0.0125 (5)	0.0349 (6)
O1W	0.0574 (6)	0.0887 (8)	0.0504 (6)	−0.0217 (6)	−0.0132 (5)	0.0287 (5)
C1	0.0783 (12)	0.0701 (11)	0.0688 (11)	−0.0096 (9)	0.0164 (9)	0.0245 (8)
C2	0.0648 (9)	0.0515 (8)	0.0351 (7)	−0.0004 (7)	0.0007 (6)	0.0076 (6)
C3	0.0542 (8)	0.0438 (7)	0.0331 (6)	0.0004 (6)	0.0002 (6)	0.0052 (5)
C4	0.0552 (8)	0.0509 (8)	0.0383 (7)	0.0027 (6)	−0.0103 (6)	0.0115 (6)
C5	0.0428 (7)	0.0516 (8)	0.0441 (7)	0.0010 (6)	−0.0069 (6)	0.0097 (6)
C6	0.0432 (7)	0.0388 (6)	0.0369 (6)	0.0018 (5)	−0.0015 (5)	0.0053 (5)
C7	0.0429 (7)	0.0545 (8)	0.0457 (7)	0.0061 (6)	−0.0028 (6)	0.0183 (6)
C8	0.0420 (7)	0.0565 (8)	0.0461 (7)	0.0018 (6)	0.0008 (6)	0.0134 (6)
C9	0.0410 (7)	0.0512 (7)	0.0401 (7)	0.0001 (6)	−0.0036 (5)	0.0090 (6)
C10	0.0428 (7)	0.0423 (7)	0.0365 (6)	0.0013 (5)	0.0017 (5)	0.0034 (5)
C11	0.0458 (8)	0.0580 (8)	0.0414 (7)	0.0048 (6)	−0.0005 (6)	0.0076 (6)
C12	0.0429 (8)	0.0741 (10)	0.0522 (8)	−0.0028 (7)	0.0048 (6)	0.0027 (7)
C13	0.0642 (10)	0.0536 (8)	0.0553 (9)	0.0076 (7)	0.0097 (7)	0.0193 (7)
C14	0.0473 (8)	0.0592 (8)	0.0513 (8)	0.0093 (6)	0.0046 (6)	0.0180 (7)

### Geometric parameters (Å, °)

**Table:**

N1—C9	1.3528 (17)	C4—C5	1.3721 (19)
N1—C6	1.4078 (16)	C4—H4A	0.9300
N1—H1A	0.8600	C5—C6	1.3946 (17)
N2—C12	1.327 (2)	C5—H5A	0.9300
N2—C13	1.329 (2)	C6—C7	1.3879 (18)
O3—C2	1.3296 (18)	C7—C8	1.3807 (19)
O3—C1	1.4422 (17)	C7—H7A	0.9300
O2—C2	1.2043 (18)	C8—H8A	0.9300
O1—C9	1.2156 (16)	C9—C10	1.5032 (18)
O1W—H1WA	0.9207	C10—C11	1.3758 (19)
O1W—H1WB	0.8877	C10—C14	1.3853 (19)
C1—H1B	0.9600	C11—C12	1.380 (2)
C1—H1C	0.9600	C11—H11A	0.9300
C1—H1D	0.9600	C12—H12A	0.9300
C2—O2	1.2043 (18)	C13—C14	1.375 (2)
C2—C3	1.4833 (19)	C13—H13A	0.9300
C3—C4	1.387 (2)	C14—H14A	0.9300
C3—C8	1.3871 (19)		
			
C9—N1—C6	127.41 (11)	C7—C6—C5	119.24 (12)
C9—N1—H1A	116.3	C7—C6—N1	124.08 (11)
C6—N1—H1A	116.3	C5—C6—N1	116.69 (11)
C12—N2—C13	116.54 (12)	C8—C7—C6	119.91 (12)
C2—O3—C1	116.54 (13)	C8—C7—H7A	120.0
O2—O2—C2	0(10)	C6—C7—H7A	120.0
H1WA—O1W—H1WB	100.2	C7—C8—C3	120.88 (13)
O3—C1—H1B	109.5	C7—C8—H8A	119.6
O3—C1—H1C	109.5	C3—C8—H8A	119.6
H1B—C1—H1C	109.5	O1—C9—N1	124.09 (12)
O3—C1—H1D	109.5	O1—C9—C10	120.49 (12)
H1B—C1—H1D	109.5	N1—C9—C10	115.42 (11)
H1C—C1—H1D	109.5	C11—C10—C14	117.50 (12)
O2—C2—O2	0.00 (10)	C11—C10—C9	123.90 (12)
O2—C2—O3	123.17 (13)	C14—C10—C9	118.56 (12)
O2—C2—O3	123.17 (13)	C10—C11—C12	118.98 (13)
O2—C2—C3	123.63 (14)	C10—C11—H11A	120.5
O2—C2—C3	123.63 (14)	C12—C11—H11A	120.5
O3—C2—C3	113.20 (12)	N2—C12—C11	123.99 (14)
C4—C3—C8	118.92 (12)	N2—C12—H12A	118.0
C4—C3—C2	118.31 (12)	C11—C12—H12A	118.0
C8—C3—C2	122.76 (13)	N2—C13—C14	123.67 (14)
C5—C4—C3	120.67 (12)	N2—C13—H13A	118.2
C5—C4—H4A	119.7	C14—C13—H13A	118.2
C3—C4—H4A	119.7	C13—C14—C10	119.26 (13)
C4—C5—C6	120.37 (12)	C13—C14—H14A	120.4
C4—C5—H5A	119.8	C10—C14—H14A	120.4
C6—C5—H5A	119.8		

### Hydrogen-bond geometry (Å, °)

**Table:**

*D*—H···*A*	*D*—H	H···*A*	*D*···*A*	*D*—H···*A*
N1—H1A···O1W^i^	0.86	2.10	2.9014 (14)	155
O1W—H1WA···N2^ii^	0.92	1.94	2.8510 (16)	169
O1W—H1WB···O2	0.89	1.96	2.8385 (14)	172

Symmetry codes: (i) −*x*, −*y*+2, −*z*; (ii) *x*+1, *y*+1, *z*−1.
